# Critical–Reflective Self-Assessment in Clinical Activities in a Dentistry Program at a Brazilian Public University

**DOI:** 10.3390/dj13070327

**Published:** 2025-07-18

**Authors:** Luís Eduardo Genaro, Aylton Valsecki Júnior, Silvio Rocha Corrêa da Silva, Elaine Pereira da Silva Tagliaferro, Fernanda Lopez Rosell

**Affiliations:** 1 School of Dentistry, São Paulo State University (Unesp), Araçatuba 16015-050, SP, Brazil; 2 School of Dentistry, São Paulo State University (Unesp), Araraquara 14801-903, SP, Brazil; aylton.valsecki-junior@unesp.br (A.V.J.); silvio.rocha@unesp.br (S.R.C.d.S.); elaine.tagliaferro@unesp.br (E.P.d.S.T.); fernanda.lopez-rosell@unesp.br (F.L.R.)

**Keywords:** self-assessment, students dental, professional competence

## Abstract

**Objectives:** This study aimed to analyze the critical and reflective self-assessment capacity of dentistry students based on two clinical courses that employ self-assessment processes as a strategy to foster autonomy in health care. **Materials and Methods:** Reflections from third- and fifth-year students were evaluated over a three-year period. The methodology sought to identify the presence of critical reflections, perceptions of strengths and weaknesses, and the formulation of improvement plans. **Results:** The results revealed low levels of continuous reflection, with only 20.0% of third-year students and 24.1% of fifth-year students engaging in reflection in at least half of their clinical activities. However, 78.7% of third-year students and 90.8% of fifth-year students completed at least one reflection during the academic year. The ability to sustain continuous critical reflection was observed in only 22.1% of the 453 students evaluated. These findings present a concerning scenario, as critical reflection is directly linked to competence in health care and informed decision-making. **Conclusions:** The study concludes that formative assessment processes requiring critical and reflective self-assessment must be broadly integrated into the curriculum to foster significant gains in the development of professional competencies.

## 1. Introduction

In recent decades, significant changes have occurred in the concepts of teaching and learning, resulting in important repercussions on the practices of educational assessment [1,2]. It is well established that the notion of knowledge as a decontextualized accumulation of information, of teaching as the mere transmission of encoded messages, and of learning as the rote reproduction of content delivered by teachers or textbooks no longer aligns with educational approaches that take learning seriously. Knowledge can no longer be viewed as static, nor as the exclusive domain of the school. The volume of information produced and rapidly disseminated through modern media is immense [2].

In this regard, Hernández [3] highlights that these changes have been recognized in most curricular proposals since the 1970s. These proposals emphasize assessment practices that are aligned with educational goals, aiming to make personal development the core of teaching and learning and enabling teachers to clearly understand what their students have learned and what they still need to learn. There is a growing need for what Pozo [4] refers to as knowledge management, where students’ education should foster autonomy, teaching them to seek, select, and critically interpret information and thus continuously advance their learning.

In this sense, education must go beyond the mere accumulation of information to promote meaningful internal transformation. As Rohden [5] reflects, “Discovering facts outside ourselves is instruction—realizing values within ourselves is education.” This perspective reinforces the need for an educational approach that not only informs but also forms, by cultivating internal values and awakening the latent potential in each individual. However, such self-management requires far more than informational mastery and related skills; it also demands an attitudinal component closely linked to the application of knowledge and abilities in achieving purposeful actions. This process of knowing how to act in response to a problem situation can be broadly associated with the concept of competence. For Roegiers [6], competence refers to “an individual’s ability to mobilize an integrated set of internalized resources to solve a family of problem situations.” It is crucial to distinguish between competence and skill: applying principles or rules routinely to familiar situations is a skill, whereas competence involves knowing how to act [7], requiring comprehension, application, synthesis, and evaluation [8].

According to Perrenoud [9], since human beings develop through their interactions with their environment, competencies are not a path but an adaptive outcome of one’s existence. Each person, in a unique way, develops competencies to solve problems and overcome challenges. Consequently, educators should first recognize their own competencies and limitations before engaging in teaching activities. If limitations are identified, they should actively seek to develop the necessary competencies [9]. Similarly, already-developed competencies must be adapted to their intended applications, which in itself mobilizes additional competencies. For students, it is essential to recognize that many competencies develop outside formal schooling through social interactions and life experiences. Given that professional training must now address shifting societal values and disciplinary knowledge, the current educational process must respect and nurture students’ multiple intelligences.

Aligned with this vision of competence development, the reflective portfolio emerges as a promising learning and assessment tool, aimed at fostering cognitive advancement [8]. In education, portfolios serve as a strategy to deepen the understanding of the teaching–learning relationship, enhancing both the students’ and teachers’ comprehension of this process and contributing to higher-quality outcomes [10].

The portfolio aligns with formative, process-based assessment. Unlike other methods, it is constructed by the student, based on principles of reflection, creativity, collaboration, and autonomy [11,12]. It connects assessment to pedagogical work in which students participate in decision-making, formulating their own ideas, making choices, and moving beyond merely following teacher or institutional directives. Thus, assessment becomes less about classification and exclusion and more about capturing individual learning. In this way, formative assessment contributes to improving educational quality [12,13].

According to Bizarro [14], the portfolio is an intrinsically individualized assessment tool that reflects its author’s learning process and fosters autonomy. This methodology encourages students to reflect critically on their practice, promoting reflective thinking and self-assessment and thereby helping them develop more effective strategies for professional practice [15]. Portfolios are increasingly important in professional education, as professional development is inseparable from personal growth, requiring the acquisition of relational, scientific, and pedagogical competencies [16,17].

Thus, the portfolio serves as a dynamic, ever-evolving tool that reflects the complexity of students’ academic journeys and fosters communication between teachers and students. It facilitates continuous collaboration and the systematic collection of data to support and diversify learning. Additionally, it promotes reflective thinking, as the process of constructing a portfolio through critical–reflective methodology encourages students to reconstruct their individual knowledge by analyzing their own performance in specific activities and identifying strengths, potential, and areas for improvement, ultimately leading to a greater awareness of their professional development [18,19].

Reflective practice thus offers an alternative to traditional instruction, which often focuses solely on external knowledge and disregards students’ internal world and values. The world of facts belongs to the ego and is the focus of instruction, whereas the world of values belongs to the self and is the true aim of education [5]. As such, reflective practice is a valuable tool for fostering deliberate, conscious attention to value creation and performance.

Based on the premise that the potential for reflection resides in each individual, Freire [20] argued that reflection should serve as a dynamic bridge between theory and practice. It is not enough to simply think and reflect; reflection must lead to transformative action. For Freire [20], reflection involves a continuous cycle of action and thought—thinking for action and thinking about action—and incorporates two additional dimensions: critique and lifelong learning. Critique involves epistemological curiosity, a transformation from naive to critical inquiry, while lifelong learning acknowledges the human condition of being perpetually unfinished [20].

Recognizing the epistemological relationship between theory and practice is thus essential, as theory alone does not transform reality without praxis, and practice is also not self-sufficient; theory and practice must be integrated [21]. Freire [22] emphasized that practice must be continuously reconstructed in alignment with theory so that “critical reflection on practice becomes a requirement of the theory/practice relationship; without it, theory may become empty rhetoric and practice mere activism.”

Such insights have advanced the development of a reflective culture in education, drawing on Dewey’s [23] Theory of Inquiry, which influenced Donald Schön’s theory of reflective practice for professional education. Schön’s framework consists of three core concepts: reflection-in-action, reflection-on-action, and reflection-on-reflection-in-action.

Reflection-in-action involves the knowledge present in professional activity—it encompasses both technical knowledge and problem-solving abilities, guiding intelligent action [24,25]. Reflection-on-action, according to Schön [26], involves retrospective mental reconstruction of an action to analyze it, offering new insights that can influence future actions. Reflection-on-reflection-in-action refers to the deliberate verbal articulation of reflection-in-action, enabling individuals to analyze and evaluate their own processes and outcomes [26].

For Alarcão [27], these three reflective processes constitute the professional’s practical thinking when facing divergent situations in practice. The processes are not independent but complementary, supporting rational, effective intervention. However, Sacristán [28] offers a different perspective, arguing that reflection-in-action offers limited opportunities for deeper thinking; thus, reflective processes should also occur during the planning and post-action phases. This conceptual framework has been continuously revisited and expanded by numerous scholars in education [20,21,27,29,30,31], who seek to better understand the impact of reflection on problem-solving.

Health policy development raises critical questions not only about identifying priority problems and solutions for vulnerable populations but also about training human resources to meet these needs in both scope and effectiveness. Special approaches are required for training, as certain populations have unique ethnic, social, cultural, economic, and historical characteristics that necessitate a broader understanding of the historical processes that shaped their realities and continue to influence their experiences today.

In this regard, the National Curriculum Guidelines (DCNs) for Dentistry programs in Brazil, officially established in 2002 [32], represent a significant milestone. These guidelines define the desired professional profile, emphasizing administration, leadership, communication, lifelong learning, decision-making, and health care. Students must develop competencies in all these areas, and universities must structure their curricula accordingly, beyond merely technical skills.

This shift has important implications for the teaching–learning process: information becomes just one element of a broader assessment framework, while subjective dimensions gain prominence in shaping professional competencies. These include character development and attitudinal processes that influence how students apply their knowledge and skills [33].

Thus, health education systems must provide sufficient and high-quality responses to reduce inequalities and promote actions that address the diverse challenges of society. Educational approaches must be grounded in an understanding of the complexity of health relationships and the interactive and integrative challenges of service delivery, which should be viewed as strategic tools for expanding health actions [34].

In this context, this study aims to assess third- and fifth-year dental students at UNESP—Araraquara School of Dentistry regarding their critical and reflective self-assessment of clinical activities conducted in public health-related courses (Preventive and Community Dentistry II and Primary Care—Record and Documentation).

Students’ capacities for self-assessment and proposal of improvements are aligned with the National Curriculum Guidelines for Dentistry [32], which emphasize the development of competencies for comprehensive care. It is important to highlight that building competencies requires targeted pedagogical interventions within the teaching–learning process. Consequently, this research adopts an action-research approach, in which the quality of students’ reflections will inform instructional adjustments by faculty, with the expectation that the results will contribute to students’ professional growth.

## 2. Materials and Methods

Specifically, self-assessment forms required by the courses Preventive and Community Dentistry II (third-year students) and Primary Care—Screening and Documentation (fifth-year students) were evaluated. These forms were completed weekly by students during their clinical activities. The analysis focused primarily on the presence of critical reflective processes regarding the clinical practice performed, including both positive aspects (strengths and potential) and negative aspects (weaknesses, insecurities), as well as the development of an improvement plan to address any identified difficulties.

To clarify the criteria observed in this study, critical reflection was defined as a process of awareness—an examination or analysis of the foundations and reasoning behind something; and an investigative attitude that rejects accepting ideas, facts, situations, values, or behaviors as obvious or self-evident without prior comprehensive examination and understanding. It refers to any reasoning process regarding facts, concepts, circumstances, or experiences that is voluntarily undertaken by the individual to draw their own conclusions. This process is linked to the cognitive ability to reason and inquire into both the external world and one’s internal state of mind and sensitivity, establishing a connection to action by creating an action plan based on interpretations of both oneself and the surrounding world.

### 2.1. Sample Characteristics

Over three consecutive years, the critical and reflective self-analysis abilities of third- and fifth-year dental students at the Araraquara School of Dentistry (FOAr) were assessed. These students were engaged in clinical activities directly associated with the educational content of the courses Preventive and Community Dentistry II (third year) and Primary Care—Screening and Documentation (fifth year). The sample included 225 third-year students (across three cohorts), each with the potential to produce 26 individual self-assessments per clinical year, and 228 fifth-year students (also across three cohorts), each with the potential to produce 28 individual reflections during the academic year, as presented in Table 1.

Upon examining Table 1, one may question the linearity of student submissions, given that students are permitted up to 25% absenteeism without failing due to attendance. Nevertheless, absences also occur during clinical activities, despite the knowledge that these activities are evaluated on a weekly basis. However, when a student is absent from a clinical session, they are required to justify their absence within their self-assessment report, providing a critical reflection on the reasons for their absence. In this way, each student’s portfolio reflects their attendance and level of engagement with clinical activities.

It was observed, however, that a small yet noticeable percentage of self-assessment reports were not completed (left blank), as shown in Table 2.

The data presented in Table 2 demonstrate students’ engagement with the self-assessment process, which forms part of the overall evaluation of their performance in clinical activities, whether acting as a clinician or as an assistant. The majority of students (93.8% of third-year students and 90.8% of fifth-year students) demonstrated an understanding of the importance of this self-analysis process in the clinical context, as evidenced by the completion of at least one critical–reflective report on their practice during the academic year. It should be noted, however, that these self-assessments also contribute to students’ performance evaluations, which likely influences their completion.

Despite students’ awareness of the evaluative role of these reports in clinical practice, a non-completion rate of 6.2% (third-year students) to 9.2% (fifth-year students) was observed, as indicated in Table 2. Systematic inquiries conducted in response to absences revealed several common reasons: one frequently cited reason was the urgency to leave the clinical setting due to subsequent commitments. Another common explanation was physical and mental fatigue, particularly after demanding clinical sessions or when students perceived their technical skills as insufficient for the task.

Although these personal factors were clearly observed, this study did not employ specific instruments to systematically evaluate how such issues may affect students’ ability to complete self-assessment tasks—especially since reflective processes require determination, focus, introspection, and the capacity to critically contextualize one’s own actions and their impacts.

### 2.2. Analytical Method

In the clinical activities of the Preventive and Community Dentistry II (third-year) and Primary Care—Screening and Documentation (fifth-year) courses at FOAr/UNESP, self-assessment forms are incorporated as part of each student’s clinical evaluation. In these forms, students are encouraged to critically reflect on their performance and to articulate their perceptions of the care provided in each clinical activity. Additionally, they are expected to develop an improvement plan to address any identified difficulties or inadequacies.

For this study, retrospective data produced by the students were analyzed. These data were anonymized and aggregated, ensuring that individual students could not be identified. The analysis of self-assessment documents was conducted only after students had completed the relevant courses, to avoid any influence on their reflections or any interference with the integrity of the assessment process. Since these forms constitute part of the official clinical monitoring process within each course, this research was conducted as an observational, analytical, descriptive, retrospective, and educational study, aimed at deepening theoretical understanding of everyday practices in clinical education. No personal or identifiable data were collected; only the information contained in the students’ self-assessment forms—which are established as institutional data within the official course syllabi—was used.

The content analysis method that was applied to the students’ reflections involved a set of systematic rules and procedures designed to allow controlled interpretation through deduction and inference. The goal of content analysis is not merely to describe the material but to extract meaningful knowledge from the data after treatment [35]. This technique balances objectivity and subjectivity, offering a valuable approach to data analysis that enables a rich interpretive process between the received message and the analytical response. In essence, content analysis is an investigative technique that systematically, objectively, and quantitatively describes the manifest content of communication, with the ultimate goal of interpretation [35].

The study focused on understanding students’ knowledge, interpersonal relationships, and self-management abilities in providing health care. A quantitative–qualitative analysis of students’ critical and reflective capacities was conducted based on their written expressions, considering several dimensions: interpersonal and intrapersonal relationships, technical knowledge and procedural mastery, recognition of strengths and potential, identification of weaknesses and insecurities, and development of improvement plans to address challenges or further enhance strengths.

To illustrate the frequency of reflective engagement across the academic year, students were grouped according to the number of reflections submitted. Specifically, students were categorized as having produced at least half or more of the expected critical–reflective reports (13 reports for third-year students and 14 for fifth-year students), with or without identification of strengths, weaknesses, or improvement plans. Additionally, intermediate reflection production was categorized (third-year: 6 to 12 reports; fifth-year: 6 to 13 reports), as well as a lower range of 1 to 5 reflections for both cohorts. Furthermore, students who did not identify any strengths, weaknesses, or improvement plans in their reports were also represented in the analysis.

It is important to note that the number of students differs between academic years, as shown in Table 1. The evaluative approach adopted in this study aimed to identify students’ evolving capacity for reflective practice over the course of their training. However, longitudinal comparisons of individual students across multiple years were not performed due to the time constraints of the study—such an approach would require at least six consecutive years of observation. For this phase of the research, the analysis focused on comparing students’ reflective capacities at the beginning (third year) and end (fifth year) of their clinical training.

Thus, the evaluation process primarily allowed for a comparison of reflective development across cohorts within the same stage of training—that is, among third-year students and among fifth-year students—with particular attention to potential growth in critical–reflective capacities as students progressed through the program.

Given that students were undergoing formal course evaluations and were in direct contact with the researcher, all analyses were conducted only after students had fully completed the relevant courses, ensuring that the research did not influence students’ routine assessments or well-being. Moreover, all data were handled and analyzed anonymously, and results were presented in aggregate form, precluding any possibility of individual identification.

This was a non-interventional study (no clinical interventions) designed solely to identify the presence of self-assessment processes. For this reason, students were not informed of the evaluative nature of the study in order to avoid introducing potential bias.

## 3. Results and Discussion

The pedagogical model adopted by the courses under study—active teaching–learning methodologies and critical–reflective self-assessments—has been in development since 2008, although the current self-assessment model was implemented in 2014. Its use is formally included in the respective course syllabi, providing support for a longitudinal evaluation of its impact on students’ cognitive development and their capacity to reflect on their own actions. This objective, however, stems from empirical observations made by the faculty over many years regarding the academic empowerment and growing autonomy of their students.

For the vast majority of students, particularly in the early stages of their training, the practice of completing critical–reflective self-assessments initially revealed difficulties in expressing and organizing their thoughts to align with this methodology and applying it effectively at the end of each clinical session. To address this challenge, the faculty implemented deliberate instructional strategies involving close supervision and formative feedback, helping students to reflect on their clinical experiences. This process was designed to provide both technical and personal parameters to assist students in identifying their strengths and potential, as well as recognizing difficulties and weaknesses encountered throughout clinical practice.

Nevertheless, the results presented below illustrate that some degree of resistance to change remains. Reflective practice is intellectually demanding, and learning to master it is even more complex and challenging, which can prove difficult for certain students—even when such practice is intended to foster competence and autonomy.

For clarity and comprehension, the results are organized by academic year, corresponding to the specific courses in which critical–reflective self-assessment and improvement planning methodologies are applied. The analysis focused on examining the relationship between the students’ year in the program and the maturity of their clinical reflections—particularly their ability to recognize strengths and weaknesses in their practice and to develop improvement plans to enhance or address these aspects of clinical performance.

Table 3, Table 4, Table 5 and Table 6 present data for third-year students, while Table 7, Table 8, Table 9 and Table 10 present data for fifth-year students. These tables illustrate students’ performance in self-assessment, indicating the number of critical–reflective self-assessments completed, the identification of strengths and/or potential, the recognition of weaknesses and/or insecurities, and the formulation of improvement plans—whether to further develop strengths or to address difficulties arising from identified weaknesses or insecurities. Together, these tables provide an overview of student reflection practices within each academic year, offering insights into their ability to analyze their own clinical practice and make informed decisions to improve performance.

The reflection parameters presented in the aforementioned tables were established based on at least half of the clinical activities planned in the respective course schedules (as referenced in Table 2). Accordingly, for third-year students, the assessment parameters were as follows: 13 or more reflective entries, 6 to 12 reflective entries, and 1 to 5 reflective entries within the academic year. For fifth-year students, the parameters were as follows: 14 or more, 6 to 13, and 1 to 5 reflective entries.

Additionally, some reports contained reflections but did not explicitly identify weaknesses or strengths, nor did they include a corresponding improvement plan. These parameters were designed to ensure that, at a minimum, students would produce high-quality, in-depth reflections for at least 50% of their clinical activities, given the cognitive and time demands of this reflective process. The tables display the number of students within each evaluative category.

Moreover, to allow for a more nuanced understanding of reflective processes and to ensure coherence with the students’ stage of professional development, the data were first analyzed and presented separately for each academic year. Subsequently, a broader analysis was conducted across the full sample of students to explore possible reasons for the observed patterns. The following sections present and discuss these findings.

### 3.1. Reflection from the Perspective of Third-Year Students

The following tables (Table 3, Table 4, Table 5 and Table 6) present data derived from the reflective reports of third-year students in the Preventive and Community Dentistry II course. These reports captured students’ critical–reflective self-assessments regarding their clinical activities, with attention to the identification of strengths and/or weaknesses and the development of improvement plans aimed at aligning clinical care with patients’ most urgent needs.

The following tables (Table 3, Table 4, Table 5 and Table 6) present the observations derived from the reflective reports of third-year students, focusing on their performance in the critical–reflective self-assessment of the clinical activities conducted in the Preventive Clinic of the Preventive and Community Dentistry II course. These reflections addressed students’ perceptions of strengths and/or weaknesses, along with the formulation of an improvement plan aimed at better aligning clinical care with patients’ most urgent needs.

The categorizations followed parameters based on the number of critical–reflective entries: 13 or more reflections (Table 3), 6 to 12 reflections (Table 4), and 1 to 5 reflections (Table 5) recorded in the clinical reports. Additionally, a comparison was made between students who demonstrated evidence of the identified reflective elements and those who did not (Table 6) throughout the academic year.

The categories presented in Table 3, Table 4 and Table 5 were constructed based on the total number of practice reflections produced by each student throughout the academic year, considering the full range of clinical activities experienced. Each student was classified into only one of the categories, according to the frequency of reflective records during the analyzed period, regardless of the isolated quality of any single entry.

Furthermore, the perceptions regarding strengths and/or weaknesses within the clinical process followed the same pattern as the reflections, with few identifications. When present, these insights were typically embedded within the student’s reflective practice. It was observed that not all students managed to identify specific strengths and/or weaknesses within their own reflections, indicating a certain superficiality in the reflective process. This aspect highlights a limitation in the quality of their reflective capacity, especially in terms of its critical nature, as they failed to identify what internal resources were mobilized during their work. Such an approach, with little or no reflection, tends toward automatism—a mechanistic and task-focused attitude—which is clearly contrary to the dynamic approach required in the process of care (Table 6).

According to Rodhen [5], this issue is explored through his critique of educational models that induce learning from the outside in, whereas true education, in his view, is an educative process that develops from within—drawing out the learner’s potential. This reinforces the idea of fostering values that guide appropriate and contextually sensitive actions.

Regarding the production of improvement plans, although at least one reflective report was produced by 81.3% of the total students analyzed (Table 6), a truly meaningful expression of these plans was observed in only 15.1% of students, who developed them for at least 50% of their clinical actions (Table 3). Other instances of such production can be noted in Table 4 (24.8%) and Table 5 (45.3%), but these figures should be interpreted relative to the number of reflections within each category. Specifically, among the 20% of students with consistent reflective processes (at least once every two clinical sessions), only 15.1% of those reflections included a coherent and appropriate improvement plan. This pattern is consistent across the other tables (Table 4 and Table 5), indicating a specific relationship between the presence of critical–reflective processes and the creation of improvement plans.

The lower incidence of improvement plans may be linked to difficulties in setting clear goals for overcoming challenges. To develop an effective improvement plan, one must at least recognize what is wrong and what needs to be addressed. In other words, students must engage with their weaknesses to overcome them and leverage their strengths to facilitate this process. Improvement plans were successfully developed following reflections in only 15.1% of cases (Table 3).

Finally, as a pedagogical practice within the Disciplines in question, students are systematically encouraged to produce reflections, even when they do not attend clinical sessions. This practice has contributed to the low percentage of students with no identifiable reflection (6.2%), though this figure reflects only the existence of reflections, not their frequency or depth. Additional indicators, such as the identification of strengths (78.7%), weaknesses (85.8%), and projections of improvement plans (81.3%), can be seen in Table 6. Within this context, it is worth noting that students tend to identify weaknesses more readily than strengths, which reflects a common human tendency to be more attuned to what is problematic or lacking than to what is empowering.

This perspective on the production of critical reflections about practice resonates with Morin’s studies on the complexity of being and doing. According to Morin [36], the development of complex thinking is essential for expanding knowledge. If thinking remains fragmented, reductionist, or mutilated, actions will follow the same path, resulting in increasingly simplistic, superficial, and disjointed knowledge. Moreover, the lack of recognition of one’s own strengths can lead to limited resources when facing emerging needs, thereby posing additional challenges to the processes of self-improvement and the development of a collaborative and autonomous professional profile.

### 3.2. Reflection from the Perspective of Fifth-Year Students

Next, Table 7, Table 8, Table 9 and Table 10 present the evaluations of fifth-year students regarding their reflective performance in relation to the development of dental care activities carried out in the Primary Care Clinic of the Triage and Documentation—Primary Care course. The data presentation for this cohort follows the same structure as the previous series. However, there is an important underlying consideration in this analysis: this is the final year of the Dentistry program, and students have already acquired nearly 90% of the technical knowledge. As a result, they possess noticeably greater technical skills compared to the third-year group, although this does not necessarily translate into psychological maturity.

Recent studies have identified behavioral patterns among young people related to the intensive use of mobile technologies. A certain level of immaturity in facing the challenges of adult life is observed, often associated with parental overprotection, which limits their exposure to everyday experiences and common risks. This dynamic contributes to emotional instability and difficulties in decision-making, affecting the development of autonomy and psychological balance.

The following tables represent this stage of training.

The evaluations of the fifth-year student group reveal that only 24.1% of the students engaged in critical reflections in at least 50% of the clinical activities performed, while 19.7% developed an improvement plan for future practices (Table 7). These are low percentages when compared to what would be expected for the development of a critical–reflective awareness. This is not to say that the other students did not reflect critically (45.2% produced between 6 and 13 reports, and 26.8% between 1 and 5 reports), but the frequency of adequate reflective records did not reach half of the clinical interventions, which suggests a certain lack of interest in this process, or even difficulty in engaging in critical reflection. This also impacts the formulation of improvement plans, which were produced by only 19.7% of students (Table 7).

An apparent evolutionary difference in this group, especially when compared to the third-year cohort, is the larger proportion of students in the intermediate range of reflective processes—that is, those who produced between 6 and 13 reports during the academic year (Table 8). This greater concentration of students can also be observed across other analytical indicators (strengths, weaknesses, and improvement plans) compared to the previous tables. This may be explained by a deeper understanding of the importance of reflective strategies in shaping a more prepared and suitable professional profile for the job market, especially regarding the pursuit of positions in public health care. Indeed, continuing education strategies and current SUS (Unified Health System) programs require professionals to be capable of critically reflecting on their actions and developing both individual and team improvement plans.

It is also possible to associate this trend with the conclusion of the professional training process and apprehension about entering the job market, given that many students do not yet feel fully prepared—a factor that encourages reflection on the future. In any case, these justifications are not sufficient to explain the lack of engagement observed in the majority of students. Critical reflection is still perceived as inconvenient and labor-intensive, a common complaint among students who feel too exhausted to reflect after complex clinical procedures.

Table 9 provides an overview of what was produced and what was not, considering the total number of fifth-year students. A very small proportion of students produced no reflections at all (9.2%), although the rate of students who failed to produce any improvement plans remains relatively high (22.8%). In terms of identifying strengths and weaknesses, the trend remains consistent—weaknesses are more easily identified (75.4%) than strengths (68.8%) within the reflections.

However, within the broader scope of professional training, a reflection on the expression of the outcomes of this self-assessment process suggests that civic formation—in terms of solidarity and responsibility toward others—is not yet achieved to the desired extent. This is likely related to the persistence of fragmented and compartmentalized curricular structures that do not incorporate reflective and creative processes in the presentation of knowledge, much less the development of competencies. Certainly, the National Curriculum Guidelines for health programs (Diretrizes Curriculares Nacionais)—which are based on competencies—aim to address this issue by promoting health through a civic perspective and by humanizing technical processes and procedures, not only through their problem-solving capacity but also through their ability to promote genuine health outcomes. In this regard, university-level training cannot shy away from adopting a civic pedagogy—one that considers the ability to organize knowledge, that is, to think [37].

### 3.3. Reflection from the Overall Perspective of the Students (Third and Fifth Years)

The analyses point to a surprising and uncomfortable reflection: few young students make use of critical thinking regarding their clinical activities, which suggests a mechanistic approach to care.

Despite being engaged in delivering “care,” which requires context-based attention to the individual’s needs to provide effective treatment, the processing of different risk elements and vulnerabilities in each case has little influence on the search for strategies that align with the complexity of the factors involved in disease production. The process of care becomes superficial.

The reasons behind this behavior are diverse and would certainly require a deeper psychoanalytic exploration, given the complexity of factors involved in human action. However, the results highlight evidence of limited empowerment and consequently low autonomy in applying the knowledge and skills acquired during the course.

Students’ perceptions of themselves are not merely linked to motivation, sense of personal efficacy, agency, or metacognition. Self-image is associated with self-confidence and the security of one’s own existence, whereby these internal resources, combined with competencies, are mobilized to respond to new and/or challenging situations. Routine problems do not activate this process but rather rely on basic skill centers. According to Bruner [38], self-confidence is a learned skill: one learns that it is possible to succeed, and that it is also possible to recover in the face of failure. This underscores the importance of resilience in managing one’s actions and the close relationship between self-image and a sense of personal efficacy.

Today’s university students generally exhibit limited resilience, and although universities aim to foster greater psychological preparedness, they also face the challenge of strengthening students’ personalities so that their technical training can truly address the real-world demands of decision-making and problem management. Current evidence shows that technical knowledge alone does not ensure adequate care aimed at promoting health, beyond simply managing signs and symptoms. This supports the thinking of Freire [22], who argued that reflection acts as a dynamic bridge between theory and practice, enabling transformative action, and of Rohden (2005), who emphasized the urgent need to cultivate internal values that allow one’s inherent potential to emerge. Morin [37] further proposes that human beings construct their own identities based on freedom and autonomy, becoming subjects in relation to others, and through self-transcendence, they surpass the immediate reality—guided by an ethical dimension that shapes their values.

Moreover, it is undeniable that greater reflection is needed on the process of reflection itself and on the development of reflective portfolios. Although reflective portfolios have proven to be valuable tools for enhancing competencies, reflection, and self-assessment, they also present challenges. The process of self-assessment and focusing on one’s own actions is unsettling, which often discourages students from engaging fully in this task.

While reflection can empower students to improve their interventions and investigative abilities, as well as conceptualize the knowledge underlying their actions, it requires discipline and determination. Once the novelty wears off, students may fall into the habit of repeating the same observations, resulting in content that is merely descriptive and adds little value. In these cases, students may view the portfolio simply as a time-consuming task that offers minimal benefit to their clinical practice. Minimizing reflection often serves as a defense mechanism to avoid confronting personal strengths and weaknesses, leading students to produce superficial accounts of their experiences.

Systematic reflection on one’s own actions should be an essential goal in developing professional capacities and competencies for health promotion. However, the study found that frequent reflective practice occurred in only a small portion of the students evaluated (100 students—22.1%), indicating that most students do not yet recognize the value of this approach in shaping their professional identities.

To address this limitation, Sá-Chaves [39] suggests avoiding repetitive reflections or descriptions that do not add value. In this regard, the present analysis disagrees, as repetition can offer important insights into students’ development, particularly in how they confront difficulties and failures—an essential aspect of learning. Interestingly, although not directly evaluated in this study, feedback from clinical seminars revealed that students viewed reflective portfolios positively and understood their role as reflective agents in constructing their own learning.

This work aims to contribute to the development of professional training grounded in values and competencies for a civic life that meets the needs of health care and promotion, as emphasized in the National Curriculum Guidelines (DCN Odontologia).

In evaluating the critical–reflective self-assessment capacities of third and fifth-year Dentistry students at the Araraquara School of Dentistry—UNESP, in clinical activities within public health-oriented courses (Preventive and Public Health Dentistry II and Triage and Documentation—Primary Care), the results point to low levels of continuous reflection—that is, reflection occurring in at least half of the clinical activities conducted during the academic year. Overall, both the third-year (20.0%) and the fifth-year (24.1%) cohorts showed modest percentages of critical reflection.

However, occasional reflections were observed in the students’ portfolios: in the third year, 78.7% of students produced at least one reflection during the year, while in the fifth year, this figure reached 90.8%. Still, the proportion of students across both cohorts (453 students total) who demonstrated deeper critical reflection was small (22.1%—100 students), painting a somewhat discouraging picture for a profession fundamentally centered on care and health promotion, where reflective processes are directly tied to competencies in care, diagnostic capacity, and conscious decision-making.

Moreover, the study concludes that formative assessment processes requiring critical and reflective self-assessment can only be successful if they are widely integrated across the curriculum. When used sporadically, as in this case, they can yield significant learning gains and contribute to personal development and competency-building for health care practice—though these benefits are seen primarily in a smaller group of highly engaged students (22.1%). For the majority (77.9%), self-analysis tends to be a response to the assessment requirements of these specific courses, rather than a self-driven practice. This stands in contrast to the desired model of critical–reflective self-assessment that supports autonomous professional attitudes and aligns with the profile envisioned in the National Curriculum Guidelines [32] for health courses, particularly in Dentistry.

This study presents an initial exploration of critical–reflective self-assessment practices among dental students; however, we recognize certain limitations pointed out by the reviewer that open important avenues for future research. The cross-sectional design, while allowing comparisons between different cohorts of third- and fifth-year students, does not permit longitudinal tracking of individual development in reflective competence. We acknowledge that following the same students over time would provide deeper insights into their reflective growth and learning trajectories.

Furthermore, the absence of a control or comparison group limits the ability to assess the specific impact of the reflective portfolio model against alternative approaches. Future studies should consider including control groups or students from institutions with different curricular structures. We also agree that the inclusion of targeted interventions—such as reflective writing workshops, peer feedback models, or structured mentoring—would enrich the learning process and could be systematically evaluated for their effectiveness. Lastly, while our study focused on aggregate data, more in-depth qualitative analyses of individual portfolios could provide richer evidence about the quality and depth of students’ reflective practice, which represents an important direction for further investigation.

## 4. Conclusions

This study reinforces the importance of integrating competency-based and reflective assessment throughout dental education to support the development of essential professional skills. Despite national guidelines emphasizing such competencies, efforts to fully implement them in curricula remain limited. Promoting critical–reflective practice continues to be a key challenge in preparing professionals capable of delivering comprehensive, autonomous, and humanized care. Sustained commitment is needed to align educational practices with these goals and foster the formation of reflective, responsible practitioners.

## Figures and Tables

**Table 1:** Number of students and potential self-assessments generated in clinical activities, by year and cohort. FOAr, 2019.

Cohort	No. of Students Involved		No. of Possible Self-Assessments in Clinics	
	3rd Year	5th Year	3rd Year	5th Year
A	75	76	1950	2128
B	76	77	1976	2156
C	74	75	1924	2100
Total	225	228	5850	6384

**Table 2:** Number and percentage of self-assessment reports completed and not completed (blank), by academic year and overall, FOAr, 2019.

Cohort	No. of Self-Assessment Reports Produced in Clinics		No. of Blank Clinical Self-Assessment Reports	
	3rd Year	5th Year	3rd Year	5th Year
A	1742 (89.3%)	1820 (85.5%)	208 (10.7%)	308 (14.5%)
B	1872 (94.7%)	1988 (92.2%)	104 (5.3%)	168 (7.8%)
C	1872 (97.3%)	1988 (94.7%)	52 (2.7%)	112 (5.3%)
Total	5486 (93.8%)	5796 (90.8%)	364 (6.2%)	588 (9.2%)

**Table 3:** Number and percentage of students with 13 or more critical–reflective entries, third-year students, School of Dentistry of Araraquara—FOAr, 2019.

Evaluated Cohort	Number of Students Evaluated	With 13 or More Critical–Reflective Self-Assessment Entries	With 13 or More Identifications of Strengths	With 13 or More Identifications of Weaknesses	With 13 or More Improvement Plans Proposed
A	75/100%	12/16.0%	8/10.7%	7/9.3%	11/14.7%
B	76/100%	15/19.7%	11/14.5%	10/13.2%	9/11.8%
C	74/100%	18/24.3%	13/17.6%	9/12.2%	14/18.9%
Total	225/100%	45/20.0%	32/14.2%	26/11.5%	34/15.1%

**Table 4:** Number and percentage of students with 6 to 12 critical–reflective entries, third-year students, School of Dentistry of Araraquara—FOAr, 2019.

Evaluated Cohort	Number of Students Evaluated	With 6 to 12 Critical–Reflective Self-Assessment Entries	With 6 to 12 Identifications of Strengths	With 6 to 12 Identifications of Weaknesses	With 6 to 12 Improvement Plans Proposed
A	75/100%	20/26.7%	19/25.3%	20/26.7%	20/26.7%
B	76/100%	21/27.6%	19/25.0%	20/26.3%	18/23.7%
C	74/100%	24/32.4%	17/23.0%	23/31.1%	18/24.3%
Total	225/100%	65/28.9%	55/24.4%	63/28.0%	56/24.8%

**Table 5:** Number and percentage of identifications with 1 to 5 indications among 3rd-year Dentistry students at Araraquara Dental School (FOAr), 2019.

Evaluated Cohort	Number of Students Evaluated	With 1 to 5 Critical–Reflective Self-Assessment Entries	With 1 to 5 Identifications of Strengths	With 1 to 5 Identifications of Weaknesses	With 1 to 5 Improvement Plans Proposed
A	75/100%	35/46.7%	30/40.0%	35/46.7%	26/34.7%
B	76/100%	36/47.4%	31/40.8%	36/47.4%	36/47.4%
C	74/100%	30/40.5%	29/39.2%	30/40.5%	30/40.5%
Total	225/100%	101/44.9%	90/40.0%	101/44.9%	92/40.9%

**Table 6:** Relationship between the number and percentage of students with or without identifications of critical–reflective processes, strengths and/or weaknesses in reflections, and projections of improvement plans among 3rd-year Dentistry students at Araraquara Dental School (FOAr), 2019.

Class Evaluated	Number of Students Evaluated	Indications of Critical–Reflective Processes		Indications of Perceived Strengths		Indications of Perceived Weaknesses		Projections of Improvement Plans	
		Yes	No	Yes	No	Yes	No	Yes	No
A	75 (100%)	67 (89.3%)	8 (10.7%)	57 (76.0%)	18 (24.0%)	63 (84.0%)	12 (16.0%)	57 (76.0%)	18 (24.0%)
B	76 (100%)	72 (94.7%)	4 (5.3%)	61 (80.2%)	15 (19.8%)	66 (86.8%)	10 (13.2%)	64 (84.2%)	12 (15.8%)
C	74 (100%)	72 (97.3%)	2 (2.7%)	59 (78.7%)	15 (20.3%)	64 (86.5%)	10 (13.5%)	62 (83.8%)	12 (16.2%)
Total	225 (100%)	211 (93.8%)	14 (6.2%)	177 (78.7%)	48 (21.3%)	193 (85.8%)	32 (14.2%)	183 (81.3%)	42 (18.7%)

**Table 7:** Number and percentage of students with 14 or more identifications in the 5th-year Dentistry course at Araraquara Dental School (FOAr), 2019.

Class Evaluated	Number of Students Evaluated	With 14 or More Indications of Critical–Reflective Self-Assessment	With 14 or More Indications of Perceived Strengths	With 14 or More Indications of Perceived Weaknesses	With 14 or More Projections of Improvement Plans
A	76/100%	16/21.1%	9/11.8%	10/13.2%	14/18.4%
B	77/100%	19/24.7%	11/14.3%	10/13.0%	15/19.5%
C	75/100%	20/26.7%	12/16.0%	15/20.0%	16/21.3%
Total	228/100%	55/24.1%	32/14.0%	35/15.3%	45/19.7%

**Table 8:** Number and percentage of identifications with 6 to 13 indications among 5th-year Dentistry students at Araraquara Dental School (FOAr), 2019.

Class Evaluated	Number of Students Evaluated	With 14 or More Indications of Critical–Reflective Self-Assessment	With 14 or More Indications of Perceived Strengths	With 14 or More Indications of Perceived Weaknesses	With 14 or More Projections of Improvement Plans
A	76/100%	34/44.7%	22/28.9%	26/34.2%	29/38.2%
B	77/100%	36/46.7%	26/33.7%	27/35.1%	30/39.0%
C	75/100%	33/44.0%	25/33.3%	27/36.0%	31/41.3%
Total	228/100%	103/45.2%	73/32.0%	128/56.1%	90/39.5%

**Table 9:** Number and percentage of identifications with 5 or fewer indications among 5th-year Dentistry students at Araraquara Dental School (FOAr), 2019.

Class Evaluated	Number of Students Evaluated	With 14 or More Indications of Critical–Reflective Self-Assessment	With 14 or More Indications of Perceived Strengths	With 14 or More Indications of Perceived Weaknesses	With 14 or More Projections of Improvement Plans
A	76/100%	15/19.7%	17/22.4%	22/28.9%	14/18.4%
B	77/100%	16/20.8%	15/19.5%	24/31.2%	12/15.6%
C	75/100%	18/24.0%	20/26.7%	20/26.6%	15/20.0%
Total	228/100%	49/21.5%	52/21.9%	66/28.9%	41/18.0%

**Table 10:** Relationship between the number and percentage of students with or without identifications of critical–reflective processes, strengths and/or weaknesses in reflections, and projections of improvement plans among 5th-year Dentistry students at Araraquara Dental School (FOAr), 2019.

Class Evaluated	Number of Students Evaluated	Indications of Critical–Reflective Processes		Indications of Perceived Strengths		Indications of Perceived Weaknesses		Projections of Improvement Plans	
		Yes	No	Yes	No	Yes	No	Yes	No
A	76 100%	65 85.5%	11 14.5%	48 63.2%	28 36.8%	54 71.1%	22 28.9%	57 75.0%	19 25.0%
B	77 100%	71 92.2%	6 7.8%	52 67.5%	25 32.5%	56 72.7%	21 27.3%	57 74.0%	20 26.0%
C	75 100%	71 94.7%	4 5.3%	57 76.0%	18 24.0%	62 82.7%	13 17.3%	62 82.7%	13 17.3%
Total	228 100%	207 90.8%	21 9.2%	157 68.8%	71 31.1%	172 75.4%	56 24.6%	176 77.2%	52 22.8%

## Data Availability

The raw data supporting the conclusions of this article will be made available by the authors on request.
